# Milk and Its Derivatives as Sources of Components and Microorganisms with Health-Promoting Properties: Probiotics and Bioactive Peptides

**DOI:** 10.3390/foods13040601

**Published:** 2024-02-16

**Authors:** Laura Quintieri, Francesca Fanelli, Linda Monaci, Vincenzina Fusco

**Affiliations:** National Research Council of Italy, Institute of Sciences of Food Production (CNR-ISPA), 70126 Bari, Italy; laura.quintieri@ispa.cnr.it (L.Q.); linda.monaci@ispa.cnr.it (L.M.); vincenzina.fusco@ispa.cnr.it (V.F.)

**Keywords:** minor dairy species, non-bovine milk, probiotics, peptides safety, nutraceuticals, foodomics

## Abstract

Milk is a source of many valuable nutrients, including minerals, vitamins and proteins, with an important role in adult health. Milk and dairy products naturally containing or with added probiotics have healthy functional food properties. Indeed, probiotic microorganisms, which beneficially affect the host by improving the intestinal microbial balance, are recognized to affect the immune response and other important biological functions. In addition to macronutrients and micronutrients, biologically active peptides (BPAs) have been identified within the amino acid sequences of native milk proteins; hydrolytic reactions, such as those catalyzed by digestive enzymes, result in their release. BPAs directly influence numerous biological pathways evoking behavioral, gastrointestinal, hormonal, immunological, neurological, and nutritional responses. The addition of BPAs to food products or application in drug development could improve consumer health and provide therapeutic strategies for the treatment or prevention of diseases. Herein, we review the scientific literature on probiotics, BPAs in milk and dairy products, with special attention to milk from minor species (buffalo, sheep, camel, yak, donkey, etc.); safety assessment will be also taken into consideration. Finally, recent advances in foodomics to unveil the probiotic role in human health and discover novel active peptide sequences will also be provided.

## 1. Introduction

Due to their content in numerous biologically active components that provide benefits to the host health, milk and milk-derived products can be considered functional foods. Indeed, functional foods are components of the diet that not only provide energy and nutrients but also positively modulate body functions, thus boosting health by reducing the risk of disease and/or by improving a given physiological response [1]. Among the nutritional and functional components of milk and its derivatives, probiotics and biologically active peptides (BAPs) play a pivotal role. Probiotics, as declared by the FAO and the WHO and confirmed by Hill et al. [2], are defined as “live microorganisms that, when administered in adequate amounts, confer a health benefit on the host”. Recently, the implications and healthy activities of probiotics against diseases such as irritable bowel syndrome, Parkinson’ disease, and prevention and treatment of allergies have also been recognized [3,4,5]. The mechanisms of action include (i) anti-inflammatory effects via suppression of proinflammatory cytokines; (ii) the modulation of gut microbiota through antagonism and inhibition of pathogen adhesion to the intestinal epithelia via production of bacteriocins, biosurfactants and short-chain fatty acids (SCFAs); (iii) enhancement of the gut barrier function of the intestinal mucosa by downregulation of low-grade mucosal immune activation, production of proteins of tight junctions and expansion of the mucus layer; (iv) development and improvement of the immunity system [3,4,5,6].

BPAs are specific protein fragments, mainly consisting of fewer than 50 amino acids, that positively affect body functions or conditions, thus influencing health. Common bioactivities of BPAs comprise several beneficial effects such as antihypertension, antioxidant, antimicrobial, antidiabetic, and anti-inflammation activities; antihypertensive properties are exerted due to the inhibition of angiotensin I-converting enzyme (ACE) and renin activities as well as the induction of vasodilation via upregulation of cyclo-oxygenase (COX), prostaglandin receptor, endothelial nitric oxide synthase expression and L-type Ca^2+^ channel blockade [7]; the relaxation of the mesenteric artery and the reduction in blood pressure in a cholecystokinin (CCK)-dependent manner has also been demonstrated for the peptide KFWGK released from bovine serum albumin (BSA) after subtilisin digestion [8]. In CaCo-2 cells, the antioxidant activity of milk-derived peptides has been attributed to the activation of the Keap1/Nrf2 pathway responsible for the overexpression of antioxidant enzymes such as glutathione reductase (GR), NADPH quinone oxidoreductase (NQO1), superoxide dismutase (SOD1) and thioredoxin reductase 1 [9]. The main mechanisms of antimicrobial peptides are instead related to changes in the physiological function of membranes and extravasation of cytoplasmic content [10]. Milk proteins can release BAPs during food processing and gastrointestinal digestion through enzymatic hydrolysis and fermentation. BPAs can also be obtained via chemical synthesis or recombinant deoxyribonucleic acid (DNA) technology of predicted active sequences [11,12]; shotgun proteomics and protein-based bioinformatics represent only an example of the current workflow for the identification and characterization of new potential food-derived bioactive peptides [13]. Likewise, the holistic effects of probiotic supplementation on inter- and extra-intestinal diseases are being demonstrated due to multiomics approaches in probiotic studies, coined “pro-biomics” [14]. 

The growing interest in milk-derived bioactive components, BPAs and probiotics is determined by the rising demand for sustainable nutraceuticals, i.e., produced by processes with high efficiency and low environmental impact, which are also safe, i.e., having a high bioavailability and none or few unwanted side effects. As consequences of this trend, the investigation of minor dairy species (buffalo, goat, sheep, mithun (*Bos frontalis*), yak (*Poephagus grunniens*) camel, donkey, and mare), counting from 11 to 0.2% of worldwide milk production, is increasing in recent years. These latter species show notable differences in composition: ruminant milk (cattle, sheep, and goats) is characterized by a high fat content and more caseins among protein fractions, while non-ruminant milk (mare, donkey) has more lactose and whey proteins content. Also the non-protein nitrogen (NPN) content (free amino acids, peptides, creatine, urea, ammonia, uric acid, orotic acid) is highly variable; for instance, the NPN content in mare milk is approximately 10–15% of the total milk nitrogen content, in cow milk, it is 5%, whereas ruminant milk has approximately 3–5% NPN [15]. Buffalo milk also has higher levels of fats, proteins, lactose, vitamin A, vitamin C and calcium than bovine milk. However, buffalo milk has a lower vitamin E and cholesterol; in addition, buffalo milk exhibits a higher buffering capacity (acidification capacity) than bovine milk. Further differences in the nutritional composition of non-bovine milk are reported by several recent studies [15,16,17]. 

Consequently, different functional health benefits have been found, e.g., compared to bovine milk, some studies suggest that milks from small ruminants (e.g., goat) cause fewer allergenic reactions due to the protein concentration and polymorphism [16,17]; similarly, conjugated linoleic acid and orotic acid in sheep milk aid the treatment and prevention of type 2 diabetes, cancer, and other diseases [16]. Non-bovine milk products are also good for isolating novel potential probiotics and probiotic carrier candidates [17]. Similarly, novel peptide sequences or peptides with improved stability, bioavailability, and efficiency could be obtained [15]. Taking into account this context, we firstly provide an overview of the detection and characterization of probiotics and BPAs in milk and milk-derived products as well as an updated review of their health-promoting properties; a careful consideration on the safety of probiotics and BPAs will also be given. In addition, we will provide an overview of the current state of the art, forthcoming challenges and tendencies of probiotics and BPAs in milk and its derivatives as well as their role in human health. Moreover, an overview of foodomics used to detect and characterize these healthy products/microorganisms will be provided. 

## 2. Probiotics in Milk and Milk-Derived Products

A growing interest towards healthy foods is emerging worldwide in recent decades, with probiotic foods attracting the highest interest for their beneficial properties exerted on human health. As a result, the research on probiotic microorganisms is growing accordingly. Although probiotics isolated from humans should be more resistant to gastrointestinal conditions, the FAO/WHO [18] reported that the action rather than the source of microorganisms makes them probiotics. Therefore, probiotics may be found not only in humans but also in other ecological niches. Milk, with its high content of nutritious compounds, is a good medium for both beneficial and detrimental microorganisms [19]. Numerous lactic acid bacteria, which are among the most used probiotic microorganisms, have been isolated from milk and their safety and probiotic potential have been assessed (Table 1). Sieladie et al. [20] assessed the safety, cholesterol-lowering properties, and antimicrobial activity of 107 lactobacilli isolated from raw milk in the Western highlands of Cameroon. Fifteen isolates were selected for bile and acid tolerance, and all showed the ability to assimilate cholesterol in vitro and bile salt hydrolase activity [20]. Almost all isolates were sensitive to eight of the nine antibiotics tested, while all showed no hemolytic and gelatinase activity [20]. Only one strain, namely isolate 29V, showed antimicrobial activity against the target pathogens. All isolates were identified as *Lactobacillus* (*Lb.*) *plantarum* (recently amended to *Lactiplantibacillus plantarum* by phenotypic methods and typed by RAPD-PCR [20]). According to the overall results, the best potential probiotic strains were *Lb. plantarum* strains 1Rm, 11Rm and 29V [20]. Banwo et al. [21] isolated and identified two *Enterococcus faecium* strains from raw milk. The strains were characterized for their technological and probiotic features and a safety assessment was carried out, finding them suitable as starters for the production of fermented foods [21]. Eid et al. [22] isolated several lactobacilli from raw cow, buffalo and goat milk and demonstrated their antimicrobial activity against mastitis pathogens. Bin Masalam et al. [23] isolated 46 lactic acid bacteria strains and assessed their safety and probiotic potential. Two *Lb. casei*, one *Lb. plantarum* and one *E. faecium* strains showed the best probiotic potential [23]. Fourteen *Lactococcus lactis* strains isolated from raw milk and kefir grains were characterized for their technological and probiotic potential, finding that the strains isolated from kefir had a higher probiotic potential than those isolated from milk, which showed the best biochemical and technological features [24]. Reuben et al. [25] assessed the probiotic potential of lactic acid bacteria isolated from indigenous Bangladeshi raw milk, investigating antagonistic activity against pathogenic bacteria, survivability in simulated gastric juice, tolerance to phenol and bile salts, auto- and co-aggregation, adhesion to ileum epithelial cells, α-glucosidase inhibitory activity, hydrophobicity, and antibiotic susceptibility, finding *Lb. casei* C3, *Lb. plantarum* C16, *Lb. fermentum* G9, and *Lb. paracasei* G10 to be the most promising probiotic bacteria.

Daneshazari et al. [26] carried out a probiotic characterization and safety assessment of *Bacillus* spp. isolated from camel milk. In particular, tolerance to acid, bile salts and artificial gastric juice was assessed, followed by auto-aggregation, cell surface hydrophobicity, antioxidant characteristics, and ability to adhere to HT-29 cells. Hemolytic and lecithinase activities were also evaluated. The *Bacillus subtilis* CM1 and CM2 strains were found to be the most promising probiotics [26].

The probiotic properties of *Bacillus subtilis* GM1, a strain isolated from goat milk, were assessed in vitro [27]. 

An ancient method to avoid the spoilage of milk, thus preserving it, is fermentation. Raw milk or thermized/boiled milk may be subjected to (i) natural fermentation, (ii) black-slopping or (iii) adjunct of commercial starter or single/multiple autochthonous microbial cultures. Depending on the raw materials used, the production step, the equipment and the manufacturing environment involved, the metabolic activity of the resulting specific microbiota is responsible for the final textural, sensorial, and probiotic features of each fermented milk and dairy product [28,29,30,31,32,33,34,35,36,37,38,39]. Within the microbiota responsible for the transformation of milk into fermented milk and dairy products, a pivotal role is played by lactic acid bacteria (LABs) while yeasts are arising as important contributors for their technological and probiotic attributes [39]. As reported in Table 1, fermented milks from cows as well as minor dairy species are an important source of probiotic microorganisms. 

**Table 1:** Potential probiotic microorganisms isolated from fermented milks ^b^ (updated from Fusco et al. [34]).

Strain	Species ^a^	Origin	Probiotic Features Tested	Reference
CYC 10058	*Lactobacillus* (*Lb.*) *kefiranofaciens*	Kefir	In vitro functional characterization: acid and bile salt tolerance, adhesion, antimicrobial activity, production, purification, and structural characterization of kefiran, inhibition of *Salmonella typhimurium* adhesion to Caco-2 cells.	[40]
SR246	*S. cerevisiae*	Spontaneous fermented cow milk, Sudan	In vitro functional characterization: low pH and bile tolerance, cytokine assay, adhesion to the non-tumorigenic porcine jejunal epithelial cell line.	[41]
IS-10506 IS-20506 IS-27526 IS-23427 IS-16183	*Lb. plantarum* *Lb. plantarum* *Enterococcus* (*E.*) *faecium* *E. faecium* *E. faecium*	Dadih, Indonesian fermented buffalo milk	In vitro functional characterization: mucus adhesion, inhibition of pathogen adhesion, displacement of pathogens, competition between pathogens and LAB strains.	[42]
CDCA 8348	*Lb. kefiri*	Kefir	In vitro ability to produce antimicrobial compounds. In vitro protective action against the invasion of *Salmonella enterica serovar* Enteritidis (*Salmonella enteritidis*). In vitro safety characterization (antibiotic susceptibility and hemolytic activity). In vitro ability to antagonize the cytotoxic effects of clostridial toxins on Vero cells. In vivo impact of CDCA 8348 oral administration for 21 days on the mucosal immune response and gut microbiota in mice. In vitro adhesion properties. In vivo proinflammatory cytokine secretion and the absence of translocation of microorganisms to blood, spleen, and liver in mice orally administered with and without CDCA 8348.	[43,44,45,46,47]
BFE6058 BFE6059	*Lb. acidophilus* *Lb. acidophilus*	Maasai fermented milk products	In vitro functional and safety characterization: resistance to low pH, bile salts deconjugation, tolerance to simulated GIT, auto-aggregation, adhesion properties to human cell lines, cholesterol assimilation, antibiotic susceptibility, DNAse activity, biogenic amine production, mucin degradation, hydrophobicity, hemolysis, antigenotoxic properties (ability to protect cells from DNA damage).	[48]
Zhang ZL12-1 BX6-6	*Lb. casei* *Lb. helveticus* *Lb. plantarum*	Koumiss	In vitro functional characterization: acid tolerance, antimicrobial activities, ability to grow in MRS with bile salts, and viability in prolonged cold storage of fermented milk.	[49]
05AM23 06TCa8 06Tca19 06Tca22 06Tca39 06Tca40 06Tca43 06TC3	*Lb. plantarum* *Lb. plantarum* *Lb. paracasei* subsp. *paracasei* *Lb. paracasei* subsp. *paracasei* *Lb. paracasei* subsp. *tolerance* *Lb. plantarum* *Lb. paracasei* subsp. *paracasei* *Lb. delbruekii* subsp. *lactis*	Airag (alcoholic Mongolian fermented mare milk) Tarag (Mongolian yogurt made from camel milk) Tarag (Mongolian yogurt made from cow milk)	In vitro functional characterization: adhesion to Caco-2 cells, resistance to acid and bile salts.	[50]
T3L	*Lb. coryniformis* subsp. *torquens*	Tibetan fermented yak milk	In vitro immunomodulating activity.	[51]
NS8	*Lb. helveticus*	Mongolian fermented koumiss	In vivo and ex vivo assessment of the anti-inflammatory attributes. In vitro functional characterization: resistance to acid and bile salts, aggregation, adhesion to Caco-2 cells, cell surface hydrophobicity.	[52]
KII13	*Lb. helveticus*	Fermented cow milk	In vitro functional characterization: tolerance to simulated orogastointestinal tract, antimicrobial activity, bile salt hydrolase activity, adhesion, and cholesterol assimilation. In vivo cholesterol-lowering activity (in mice).	[53]
S1K3 (MTCC5957)	*Lb. rhamnosus*	Indian fermented milk	In vitro functional characterization: antagonistic activity, adhesion, protective effect against *Salmonella enterica*. In vivo assessment of the protective effect against *Salmonella enterica* in mice after long-term (30 days) consumption of S1K3-fermented milk.	[54]
M1	*Lb. kefiranofaciens*	Taiwanese kefir grains	In vivo and in vitro anti-colitis, anti-asthmatic, antiallergic, and immunomodulatory effect; in vivo oral toxicity. Effects of heat, cold, acid bile salt adaptations on stress tolerance and protein expression.	[55,56,57,58,59,60]
DN1	*Lb. kefiranofaciens*	Kefir	In vivo modulation of gut microbiota and increase in fecal water content in mice induced by administration of DN1.	[61]
Lp3	*Lb. plantarum*	Fermented Tibetan yak milk	In vitro functional characterization: cell surface hydrophobicity, bile tolerance, and survival in simulated GIT. In vitro cholesterol-lowering test. In vivo cholesterol-lowering potential in rats.	[62]
Jlus66	*Lb. paracasei*	Chinese naturally fermented milk	In vitro functional and safety characterization: tolerance to simulated GIT, in vitro cholesterol-lowering activity, cell surface hydrophobicity, and hemolytic activity. In vivo probiotic characterization: effect of the probiotic strain on non-alcoholic fatty liver in rats.	[63]
Pro4 Pro7	*Lb. paracasei* *Lb. rhamnosus*	Raw and fermented camel milk	In vitro probiotic and safety characterization: phenol, bile salts, sodium chloride and acid tolerance, antimicrobial activity, antibiotic susceptibility, and hemolysis. In vivo assessment of the mucosal immune response in mice.	[64]
DD2	*Lb. kefiranofaciens*	Kefir	In vitro survivability in an experimental oral environment, antimicrobial activity, and antibiofilm formation activity against *S. mutans* and *S. sobrinus*.	[65]
C4	*Lb. plantarum*	Kefir	In vitro evaluation of the fermentation properties and potential probiotic activity in batch culture systems. Physicochemical, nutritional, and organoleptic characterization of a skimmed goat milk fermented with C4. In vivo immunomodulatory effect in mice.	[66,67,68]
KCTC 5075	*Lb. kefiranofaciens*	Kefir	In vivo assessment of the therapeutic effects of extracellular vesicles derived from KCTC 5075 in mice with 2,4,6-trinitrobenzene sulfonic acid-induced inflammatory bowel disease.	[69]
K2	*E. faecalis*	Kalarei, Indian fermented milk	In vitro probiotic and safety characterization: bile salt deconjugation, cholesterol assimilation ability, cholesterol-removing potential, SEM analysis for adsorption of cholesterol to probiotic cell surface, antioxidant potential, hemolytic activity, gelatin-hydrolyzing activity, biogenic amine production, and antibiotic susceptibility.	[70]
YS5	*Lb. plantarum*	yogurt	In vitro and in vivo cholesterol assimilation. In vitro probiotic and safety characterization: acid and bile salt tolerance, bile salt hydrolase activity, antimicrobial activity, hemolysis, and antibiotic susceptibility.	[71]
MSR101	*Lb. kefiri*	Chinese kefir grains	Characterization and in vitro antitumor activity of exopolysaccharide produced by the strain.	[72]
N16	*Lactobacillus* spp. ^a^	Indonesian dadih (fermented buffalo milk)	In vitro functional characterization: acid and bile salt tolerance, adhesion to mucosal surface and antagonism against enteric pathogens.	[73]
SL2, SL3 and SL5 SN1, SN7, SN8 and SN9	*Saccharomyces cerevisiae* *S. cerevisiae*	Lait caille Nunu (African fermented cattle milks)	In vitro functional characterization: resistance to acid and bile salts, effect of yeasts on integrity of the Caco-2 cell monolayers, adhesion, and changes in pH in yeast cells during perfusion with the gastrointestinal pH.	[74]
GCC_19M1	*Lb. plantarum*	Indian fermented raw milk	In vitro probiotic and safety characterization: resistance to acid, sodium chloride, bile salts and pancreatin, tolerance to gastric juice model auto-aggregation, cell surface hydrophobicity, glucose fermentation, antimicrobial activity, antibiotic susceptibility, and hemolysis.	[75]
SD11	*Lb. rhamnosus*	Fermented milk	In vivo functional characterization: effect of fermented milk containing *L. rhamnosus* SD11 and maltitol on *S. mutans* in a double-blind, randomized, controlled study in humans.	[76]
IS-10506	*Lb. plantarum*	Dadih (Indonesian fermented water buffalo milk)	In vivo probiotic characterization: intestinal stem cell activation to counter inflammation in a rodent model. Level of blood lipopolysaccharide and immune response in HIV-infected children. Faecal secretory immunoglobulin A level and immune response in children younger than two years. Effect of IS-10506 and zinc supplementation on humoral immune response and zinc status of Indonesian pre-school children. Effect on the Scoring Atopic Dermatitis Index (SCORAD) in children with atopic dermatitis. Renal tubular regeneration in pyelonephritic rats.	[77,78,79,80,81,82]
BIOTEC006 BIOTEC007 BIOTEC008 BIOTEC011 BIOTEC012 BIOTEC013 BIOTEC014 BIOTEC015 BIOTEC016	*Lactococcus* (*Lc*.) *lactis* *Lc. lactis* *Lc. lactis* *Leuconostoc* (*Leuc.*) *pseudomesenteroides* *Leuc. pseudomesenteroides* *Lentilactobacillus* (*Lentil.*) *kefiri* *Lentil. kefiri* *Lentil. parakefiri* *Lc. lactis*	Mexican milk kefir grains	In vitro probiotic characterization: resistance to simulated GIT, antimicrobial activity, aggregation, antibiotic susceptibility, and GABA production fermentability with commercial prebiotics.	[83]
MK Y55 MK L1	*Kluyveromyces marxianus* *Lc lactis*	Raw fermented milk	In vitro functional characterization: acid and bile salt tolerance, lactose utilization, resistance to simulated GIT, manufacture of fermented milk using the probiotic strains and microbial viability during storage, and resistance to simulated GIT of microorganisms in fermented milk.	[84]
NWAFU-BIO-BS29	*Lactiplantibacillus plantarum*	Chinese fermented cow milk	In vitro and in vivo probiotic and safety characterization: antibacterial activity, acid and bile salt tolerance, auto- and co-aggregation, cholesterol-lowering ability, antibiotic susceptibility, hemolysis, detection of resistance and virulence genes, antioxidant activity, short-chain fatty acid production, effects of *L. plantarum* NWAFU-BIO-BS29 on body weight, organs index, and colon H&E in BALB/c mice.	[85]
NWAFU-BIO-BS29 NWAFU-BIO-AS16 NWAFU-BIO-D-S7	*Lactiplantibacillus* (*Lactip.*) *plantarum* *Companilactobacillus crustorum* *Lb. gallinarum*	Chinese fermented milk	In vitro functional and safety characterization: acid and bile salt and hydrogen peroxide tolerance, antimicrobial activity, auto- and co-aggregation, antioxidant activity, cholesterol-lowering ability, antibiotic susceptibility, hemolysis, detection of resistance and virulence genes, short-chain fatty acids analysis.	[86]
EGER41	*Lactip. plantarum*	Amabere amaruranu, Kenyan fermented milk	In vitro functional and safety characterization: phenol and acid tolerance, antagonistic activity, antibiotic susceptibility, and hemolytic activity.	[87]
As21	*Lactip. plantarum*	Fermented yak milk	In vitro probiotic characterization: hydrogen peroxide tolerance, scavenging activity of diphenylpicrylhydrazyl (DPPH) free radicals, hydroxyl radical scavenging activity, superoxide anion clearance, total antioxidant capacity and superoxide dismutase activity, cell surface hydrophobicity, bile tolerance, tolerance to simulated GIT, antioxidation and ageing experiments in worms, and probiotic effects on the oxidative senescence of *Caenorhabditis elegans*.	[88]
BB101 02	*Lb. fermentum* *Lb. casei*	Fermented milk	In vitro functional and safety characterization: acid and bile tolerance, hemolysis and antibiotic susceptibility. In vivo antiulcerogenic potentials of the probiotic strains in ethanol-induced gastric lesions model in mice.	[89]
3 strains 2 strains 15 strains	*Lc lactis subsp. cremoris* *Leuc. mesenteroides* subsp. *jonggajibkimchii* *Leuc. mesenteroides*	Indian fermented cow milk dadih Indian fermented yak milk dadih Indian fermented cow milk soft churpi Hard-variety Indian fermented yak milk *chhurpi* Indian fermented cow milk mohi Indian fermented yak milk philu	In vitro functional characterization: acid, bile and lysozyme tolerance, β-galactosidase activity, hydrophobicity, deconjugation of bile salts, and probiotic genes screening.	[90]
LB12	*Lacticaseibacillus paracasei*	Doogh, Iranian fermented milk	In vitro probiotic and safety characterization: resistance to simulated GIT, acid and bile salt tolerance, auto- and co-aggregation, cell surface hydrophobicity, adhesion capacity, antagonistic activity, DNase activity, antibiotic susceptibility, and hemolytic activity.	[91]
QAUBL19 QAUBSS1	*Bacillus licheniformis* *Bacillus subtilus*	Dadih, Pakistan fermented milk	In vitro probiotic and safety characterization: survival in simulated GIT, tolerance to acid and bile salts, antibacterial activity, antibiotic susceptibility, cell surface hydrophobicity, auto-aggregation, exopolysaccharide production, bile salt hydrolase activity, in vitro cholesterol removal activity, carbohydrate utilization ability, decarboxylase activity, antioxidant activity, hemolysis, and DNase activity.	[92]
AcCh91	*Levilactobacillus brevis*	Indian fermented milk	In vitro functional and safety characterization: acid and bile tolerance, auto- and co-aggregation, antimicrobial activity, microbial attachments to hydrocarbons, bile salt hydrolysis. β-galactosidase activity, exopolysaccharide production, cholesterol reduction, hemolysis, screening of probiotic and functional genes, and in silico analysis for safety evaluation.	[93]

^a^ The genus *Lactobacillus* has been reclassified into 25 genera Zheng et al. [94]. In this table, both the old and the new nomenclature are provided. ^b^ Where not specified, the milk used for the production of dairy products is cow’s milk.

The probiotic lactic acid bacteria most frequently found in fermented milks are strains of the recently amended *Lactobacillaceae* family [94]. Apart from lactobacilli, strains of the *Pediococcus* genus mainly belonging to *P. pentosaceus* and *P. acidilactici* species are arising for their probiotic attributes (Table 1) [95]. Among yeasts, certain strains of *Saccharomyces cerevisiae* have been found to possess probiotic attributes (Table 1). However, certain clinical and foodborne *S. cerevisiae* strains are arising as opportunistic pathogens so that, as established by the EFSA (who confirmed its Qualified Presumption of Safety, QPS status), the inability to grow above 37 °C and to resist to antimycotics compounds used in human medicine must be demonstrated prior to adding viable cells of strains of this species in the food and feed chain [39]. 

In Table 2 are listed the probiotic microorganisms isolated from milk-derived products other than fermented milks.

Once again, lactobacilli are the most prevalent followed by strains of *Enterococcus* spp. However, mainly due to their ability to acquire virulence factors and resistance to several classes of antibiotics as well as their occurrence as opportunistic pathogens, enterococci are not generally recognized as safe (GRAS) microorganism and neither do they have QPS status [165,166]. Thus, their use as probiotics is highly controversial. A similar discussion can be had for yeasts such as *Kluiveromyces marxianus* (whose anamorph name is *Candida kefir*), which was declared as a significant opportunistic pathogen by the EFSA [39] despite the probiotic attributes found in certain strains isolated from dairy products (Table 2); however, its QPS status has been confirmed [167].

As depicted in Table 1 and Table 2, studies dealing with the probiotic and safety assessment of microorganisms isolated from fermented milk and dairy products are increasing in recent years; and apart from cow milk, non-bovine milks such as buffalo, ewe, goat, yak and camel milk derivatives are important sources of probiotics and promising carriers of probiotics [168,169,170,171,172,173]. Indeed, although bovine milk still dominates the probiotic market worldwide, there is an increasing trend towards the use of milk from species other than cows to deliver probiotics. This is mainly due to the adequate shelf-life viability of probiotics as well as the intrinsic functionality of non-bovine milk. However, given the absence or low amount of kappa casein, which negatively affects their coagulation capability, camel and donkey milk are mainly used as such for probiotic delivery and only seldom for the production of probiotic cheese, whereas ewe, goat, yak and buffalo dairy products are being frequently used as carriers of probiotics. 

As for the beneficial effect of probiotics isolated from milk and milk-derived products, it should be highlighted that, although mandatory, only few articles assessed the probiotic features by in vivo studies focusing only on in vitro tests. Moreover, although a thorough safety assessment should target aspects such as antimicrobial susceptibility, metabolic activity, toxin production, side effects in humans, hemolytic activity, adverse outcomes in consumers, and infectivity in immunocompromised animal models [174], too high a number of the studies listed in Table 1 and Table 2 only carried out the antibiotic susceptibility assessment or did not perform any safety assessment at all. In addition, considering that the beneficial effect of a given probiotic is strain and dose dependent, the species- and strain-level identification of the potential probiotic as well as the enumeration of viable probiotics in a given probiotic food or supplement are mandatory to characterize probiotic microorganisms and authenticate probiotic food/beverage/supplements [175,176,177]. Moreover, the technological features of the potential probiotic should be characterized and their survival in the processing, storage, distribution, and shelf-life within the probiotic food/beverage/supplement should be assessed [176]. Nevertheless, as reported in Table 1 and Table 2, few studies made these assessments. Moreover, apart from a few articles that carried out in vivo studies in rats or in mice (Table 1 and Table 2), only one article [76] (Table 1) assessed the beneficial effects of probiotics via double-blind, randomized, controlled study in humans.

## 3. Beneficial Effects of Probiotic Milk and Milk-Derived Products

As we mentioned in the previous paragraph, no clinical trials in humans but studies using in vitro cell cultures or animal models have so been far carried out for probiotic milk and milk-derived products containing autochthonous potentially probiotic microorganisms. The pivotal role of probiotics in human health is well known [178] but controlled validated clinical trials are mandatory to verify that the health benefits are not altered or lost when the probiotic is incorporated into the food matrix due to the technological stresses it undergoes during manufacturing. The efficacy of probiotic milk and milk-derived products must be demonstrated in controlled validated clinical trials to prove that the probiotic features are not altered or lost passing from in vitro to in vivo studies. But even animal studies maybe not be adequate predictors of human experiences, humans being quite diverse from animals in terms of lifestyles, diet, and gut microbiome. However, clinical trials of probiotic milk and milk-derived products containing commercial probiotics or probiotics isolated from food matrices other than milk-based products have been carried out demonstrating numerous health benefits [179,180,181,182,183,184], leading to hypothesize that milk and dairy food/beverages containing autochthonous probiotic microorganisms would also positively impact human health. However, it should be highlighted that even numerous health claims associated with many probiotic strains already available on the market have been rejected by the EU due to either (i) insufficient characterization, (ii) invalidity of claims/unproven claims, (iii) the absence of beneficial effects on nutrition and or lack of progress on the physiological state of the body, (iv) lack of scientific basis and/or low quality of studies, and (v) the absence of placebo-controlled, double-blind clinical trials.

## 4. Bioactive Peptides from Milk Proteins: Overview of Health Benefits and Their Applications

Milk proteins are valuable sources of BPAs, exerting several health-promoting activities; well-known biological activities includes antimicrobial, antibiofilm [185,186,187], anticancer [188] anti-inflammatory [9], antihypertensive [189], antioxidative [190], opioid-like [191], dipeptidyl peptidase IV (DPP-IV), antiviral [192], and mineral-binding ability [190]). Antimicrobial and antibiofilm peptides from milk proteins display a wide spectrum of activity against bacteria and fungi with slower development of resistance mechanisms [187]; anticancer peptides induce distortion of proteins responsible for cancer cell proliferation, a reduction in the enzymatic activities associated with cancer growth, inhibition of the angiogenesis and initiation of necrosis or apoptosis processes. These mechanisms were proved for bovine or non-bovine milk-derived BPAs [193]. Opioid peptides are selective to μ-type (e.g., β-casein f (60–70) YPFPGPIPNSL), δ-type (e.g., α_S1_-casein f (90–96), RYLGYLE) and κ-type opioid receptors [191]. Recently, Wu et al. [194] also reviewed the potential of milk-derived BAPs affecting the gut microbiome, in turn regulating gut–brain functioning, gut health, and immune system activity.

BPA sequences, lower than 6 kDa, consist of 2 and 20 amino acid residues; BPAs remain inactive in the native protein and exhibit their activity after release and transport to the active site [190]. The biological properties of BPAs are affected by several factors: sequence length, amino acid motifs, and structure (α-helix, β-sheet, β-turn, and random coil); for example, the presence of alanine, cysteine, histidine, lysine, leucine, methionine, proline, valine, tryptophan, and tyrosine may contribute to antioxidant properties [195], while proline, lysine and aromatic amino acid residues contribute to ACEI activity [189,195]; branched-chain amino acid (BCAA)-rich motif sequences show high stability and antimicrobial and anticancer activities [195]. Secondary and tertiary structures are instead responsible for BPA antioxidant properties [196].

Some BPAs, in particular tripeptides, shared sequences among the same protein from different milks (goat, sheep, or camel) [197]; however, changes in BPA activity derived from proteins of different mammals can also be expected depending on the degree of homology between parent proteins [190,198].

Chronic diseases, such as metabolic diseases, have become a worldwide public health issue and several efforts are ongoing to discover novel therapeutic strategies. Overall, most published studies on rodent and cells or including clinical trials to evaluate the effects of BAPs on different pathological conditions were reported for bovine milk-derived BAPs [199,200,201,202]. To the best of our knowledge, there is, instead, limited literature about the biological effects of milk from minor species [203]. Thus, in an attempt to shed light on novel active sequences, Table 3 reports BPAs identified from native proteins of milk from minor dairy species; effective dosages determined in experimental trials, in vitro or in vivo models, were also reported. By contrast, presumptive active sequences by in silico approaches and or not validated in experimental trials were not included [204,205,206]. Overall, most identified bioactive peptide sequences from milk proteins of minor species belonged to caseins (CNs), in particular from α_S2_-casein (α_S2_-CN) and β-casein (β-CN); among whey proteins, bioactive peptides were instead released from β-lactoglobulin (β-LG). 

The inhibition of the ACE, which converts angiotensin I to the vasoconstrictor agent angiotensin II by lowering blood pressure in cardiovascular disease, is widely reported for the listed peptides (Table 3). Among biological effects, peptides from camel milk affected both glucose transport and metabolism and the structural and functional properties of the pancreatic β-cells and insulin secretion. The main targeted enzymes are major carbohydrate digestive enzymes, i.e., the pancreatic α-amylase and the intestinal α-glucosidase responsible for the digestion of almost 90% of dietary carbohydrates, in turn releasing an abundance of glucose, causing postprandial hyperglycemia. Another enzyme that has been targeted for its inhibition by BAPs is the DPP-IV enzyme, which degrades major incretin hormones involved in the release of insulin in response to glucose [207].

After their release from native protein, BAPs were usually purified, identified, and chemically synthesized to validate the presumptive activity; effective doses were also determined by assaying HPLC fractions of hydrolyzed proteins [208,209,210]. Although BAPs have attracted increasing interest in the development of functional foods or novel drugs, the production of milk-derived BAPs as well as their purification has been limited by their high cost and lack of suitable large-scale technologies. This gap has heavily compromised the application of peptides in commercial products; the crude hydrolysate solution, indeed, is a complex mixture consisting of BPAs and many non-bioactive hydrolyzed compounds. Each peptide shows different charges, physicochemical properties, and molecular weight, making purification an ongoing challenge [211]. Several separation techniques have been employed to purify such peptide mixtures (ultrafiltration, chromatography-based methods, capillary electrophoresis and recently electrodialysis–ultrafiltration) that, however, are mainly used at the lab scale [211]. Thus, commercial food products supplemented with milk protein-derived BPAs are few and mainly consist of bovine milk-derived peptides (e.g., Calpis, Calpis Co., Ltd., Tokyo, Japan; EVOLUS, Valio, Ltd., Helsinki, Finland; Lowpept, nnaves S.A., Pontevedra, Spain; BioZate 1, Davisco Foods, LeSueur, MN, USA; [212,213,214]). Ayyash et al. [215] performed a comparative study of the healthy properties of camel milk and bovine milk fermented with camel milk probiotic strain. Although the authors did not investigate the proteolytic pathways of the indigenous LABs or identify the BAPs derived from the fermentation process, the study demonstrated that the health-promoting benefits (antioxidant, ACE inhibition, and antiproliferative activities) of water-soluble extracts in fermented camel milk were markedly higher than those in fermented bovine milk [215]. A novel food application of peptides from minor dairy species was investigated by Hajian et al. [216]. In this study, a camel milk low-fat ice cream with various concentrations of antioxidant casein hydrolysates was produced; the modified recipe of ice cream improved the melting resistance of the products and registered good acceptability by panelists.

Thus, even though much is known about the peptide sequences with biological effects from milk proteins, more research is still required on novel BAPs from minor dairy species and validation of their mechanisms of action. After digestion, BPAs can be absorbed in the intestine and enter the blood stream directly by ensuring their bioavailability in vivo and their activity at the target site or can be further hydrolyzed, thus reducing their activity; for these reasons, both for BAPs from bovine and other milk species, preclinical and clinical studies are needed to determine which levels are beneficial for health, their dose–response relation, stability, bioavailability and regulatory factors, pharmacokinetics, and residual activity after ingestion in foods.

**Table 3:** List of bioactive peptides (BPAs) from milk of minor dairy species and related beneficial effects.

Origin	Process	Peptide Sequence	Native Protein	Bioactivity	Dose	References
**Camel**	Hydrolysis with alcalase or papain	KDLWDDFKGL MPSKPPLL	n.s.	α-amylase inhibitory peptides	IC_50_: 0.027, 0.025 mg/mL (as RP-HPLC fractions)	[210]
	Hydrolysis with pepsin–pancreatin	LEEQQQTEDEQQDQL YLEELHRLNAGY RGLHPVPQ	n.s.	Antioxidant	IC_50_: 0.08–0.19 mg/mL	[217]
	Hydrolysis with pepsin–pancreatin	NEDNHPGALGEPV KVLPVPQQMVPYPRQ	αs_1_-CN *f* (153–164) β-CN *f* (185–197)	Antioxidant	DPPH radical activity: 0.04, 0.02 mg/mL; ABTS radical scavenging: 0.1, 0.01 mg/mL	[218]
	Trypsin digest	RLDGQGRPRVWLGR TPDNIDIWLGGIAEPQVKR VAYSDDGENWTEYRDQGAVEGK	n.s.	Antioxidant	0.6–2.4 mg/mL	[219]
	In vitro gastrointestinal digestion	LPQ	Various proteins	DPP-IV inhibitory peptides	IC_50_: 82 µM	[197]
	Hydrolysis	LPVPQ WK	β-CN *f* (172–177) αs_2_-CN *f* (69–70)	DPP-IV inhibitory peptides	IC_50_: 43.69 µM 40.6 µM	[220]
**Camel**	Hydrolysis with trypsin	LPVP, MPVQA	β-CN *f* (172–176) β-CN *f* (186–190)	DPP-IV inhibitory peptides	87.0 ± 3.2, 93.3 ± 8.0 µM	[205]
	Hydrolysis with trypsin	VPV, YPI and VPF	β-CN	DPP-IV inhibitory peptides	IC_50_: 6.6, 35.0, 55.1 µM	[205]
	Fermentation	MVPYPQR	β-CN *f* (177–183)	Antioxidant ACEI	IC_50_: 89, 33 µM IC_50_: 30 µM	[205,221]
	Cheese	LQKW LLF	β-LG *f* (58–61) β-LG *f* (103–105)	ACEI	IC_50_: 3.5 μM IC_50_: 82.4 μM	[222]
	Milk fermentation	AIPPKKNQD	κ-CN *f* (107–115)	ACEI	IC_50_: 19.9 μM	[223]
	In vitro gastrointestinal digestion of camel milk	IPP LPP VIP	k-CN β -CN α_S2_-CN	ACEI	IC_50_: 5 μM IC_50_: 9.6 μM IC_50_: 26 μM	[197]
**Goat**	In silico prediction and in vitro validation	SWMHQPP QSLVYPFTGPIPNSL YPYQGPIVL	n.s.	Antioxidant	IC_50_: 16.83, 24.79, 20.94 µg/mL	[224]
	Kefir	LHLPLP HLPLP DKIHP	α_s2_-CN *f* (26–31) α_s2_-CN *f* (27–31) β-CN *f* (47–51)	ACEI	IC_50_: 5.5 μM 21 μM 233 μg/mL	[225]
	Milk and cheese	TVDQHQ	α_S2_-CN *f* (182–187)	ACEI	210 μg/mL	[198,226]
	Cheese	LVYPFPGPIPN LVYPFPGPIPNSLPQNIPP	β-CN *f* (58–65) β-CN *f* (58–73)	ACEI	27.9 μM 5 μM	[226]
	Hydrolysate kefir	TGPIPN LVYPFTGPIPN	β-CN *f* (78–83) β-CN *f* (73–83)	ACEI	316 μM 27.9 μM	[227]
	Hydrolysate and cheese	SLPQ	β-CN *f* (84–87)	ACEI	330 μM	[227]
	Hydrolysate and cheese	SQPK	β-CN *f* (181–184)	ACEI	354 μM	[227]
	Gastric digestion of isolated whey proteins and caseins of milk	PEQSLACQCL QSLVYPFTGPI ARHPHPHLSFM	β-LG *f* (113–122), β-CN *f* (56–66) κ-CN *f* (96–106)	ACEI	4.45 μM 4.27 μM (as mixture)	[228]
	In vitro gastrointestinal digestion	IAV VPP GPV	α_S1_-CN β -CN β -CN	ACEI	27 μM 9 μM 4.7 μM	[197]
**Goat**	In vitro gastrointestinal digestion	IPI	k-CN	DPP-IV-inhibitory activity	3.5 μM	[197]
	Milk casein hydrolysates	SDIPNPIGSE NPWDQVKR, SLSSSEESITH, QEPVLGPVRGPFP	α_S1_-CN *f* (195–204) α_S2_-CN *f* 123–130) β-CN *f* (30–40) β-CN *f* (207–219).	Anti-diabetic activity	0.50 mg/mL (as hydrolysate fractions)	[229]
	Trypsin/chymotrypsin hydrolysates	INNQFLPYPY MHQPPQPL	κ-CN *f* (51–60) β-CN *f* (144–151)	DPP-IV-inhibitory activity	40 μM 350 μM	[230]
	Hydrolysis with neutral and alkaline proteases	VYPF FPYCAP FGGMAH YPPYETY YVPEPF	n.s.	Antioxidant	83–107 µg/mL (as GFC fractions)	[209]
**Donkey**	Endogenous peptides	REWFTFLK MPFLKSPIVPF	Serum amyloid A protein *f* (19–26) *Equus caballus* (horse) β-CN *f* (130–140)	ACEI	0.71 10.3 μM	[15,231]
	Endogenous peptides	WFTFLKEAGQGAKDMWR GQGAKDMWR	Serum amyloid A protein *f* (11–29) *Equus caballus* (horse) Serum amyloid A protein *f* (20–29) *Equus caballus* (horse)	Antioxidant	16.8 μM 22.7 μM	[15,231]
**Yak**	Hydrolysis with trypsin and alcalase	TPVVVPPFL	β-CN *f* (90–100)	Anti-breast cancer	cell viability inhibition rate IC_50_: 250–500 μg/mL	[232]
	Pepsin hydrolysis	KVISMI RVMFKWA	n.s.	Antimicrobial against *Bacillus subtilis*, *Staphylcoccus aureus*, *Listeria innocua*, *Escherichia coli*, *Enterobacter cloacae*, *Salmonella paratyphi* and *Candida albicans*, *Saccharomyces cerevisiae*	MIC: 4–32 μg/mL MIC: 8–32 μg/mL	[233]
**Yak**	Two-step enzymatic hydrolysis with trypsin and pepsin	KALNEINQF	α_S2_-CN	Antioxidant	25–100 μg/mL	[234]
	Two-step enzymatic hydrolysis with trypsin and pepsin	MHQPHQPLPPTVMF	β-CN	Antioxidant	6.25, 12.5, 25, 50 μg/mL	[235]
	Fermentation by *L. plantarum* JLAU103	LYLKPR	unknown	Neuroprotective effects on H_2_O_2_-injured HT-22 cells	100–200 μM	[236]
	Hydrolysis with alcalase	RELEEL	β-CN *f* (1–6)	Superoxide anion and hydroxyl radical scavenging activity	IC_50_: 0.52 0.69 mg/mL	[237]
	Hydrolysis with trypsin, pepsin, alcalase, flavourzyme, papain and neutrase	YQKFPQY LPQNIPPL SKVLPVPQK LPYPYY FLPYPYY	α_S2_-CN *f* (89–95) β-CN *f* (70–77) β-CN *f* (168–176) κ-CN *f* (56–61) κ-CN *f* (55–61)	ACEI	IC_50_: 380 μM	[238]
	Hydrolysis with alcalase	PPEIN PLPLL	κ-CN *f* (156–160) β-CN *f* (136–140)	ACEI	IC_50_: 290 μM IC_50_: 250 μM	[238]
**yak**	Trypsin digestion combined with QSAR molecular docking	KYIPIQ	κ-CN *f* (45–50)	ACEI	IC_50_: 7.28 μM	[239]
	Hydrolysis with thermolysin/alcalase and thermolysin/proteinase K	FPQY MPFPKYP MFPPQ QWQVL	α_S2_-CN *f* (91–95) β-CN *f* (109–115) β-CN *f* (156–160) κ-CN *f* (75–79)	ACEI	IC_50_: 12.4 2.9 40.8, 112 μM	[240]
**Sheep**	Cheese	LKKISQ VRYL YIPIQY LVYPFTGPIPN	α_s2_-CN *f* (165–170) α_s2_-CN *f* (205–208) κ-CN *f* (25–30) β-CN *f* (58–68)	ACEI	IC_50_: 2.6 μM 24.1 μM 10 μM 27.9 μM	[241,242]
	Milk, kefir	PYVRYL	α_s2_-CN *f* (203–208)	ACEI, antioxidant	2.4 μM nd	[242,243]
	Cheese	ALPMHIR IIVTQTMK IDALNENK	β-LG *f* (142–148) β-LG *f* (1–8) β-LG *f* (84–91)	ACEI	IC_50_: 62.6 μM IC_50_: 70.8 μM IC_50_: 71.2 μM	[198]
	Cheese	VMFPPQSVL VVAPFPEV	β-CN *f* (155–163) α_s1_-CN *f* (24–31)	Antimicrobial activity against Gram-positive and Gram-negative bacteria	25–110 µg/mL	[244]
	In vitro gastrointestinal digestion	IPA	various proteins	DPP-IV-inhibitory activity	49 μM	[197]
**Buffalo**	Hydrolysis of whey with *Dregea sinensis* Hemsl. protease	DQPFFHYN YSPFSSFPR	α-2-glycoprotein 1, zinc-binding clustering	Anti-inflammatory activity	50–200 µg/mL	[245]
	Hydrolysis with papain	GPFPIIV YPVEPFT	β-CN	ACEI	9.1 µg/mL (as fraction)	[208]
	Hydrolysis with trypsin and pepsin	EDVPSER NAVPITPTL VLPVPQK HPHPHLSF	α_s1_-CN *f* (84–90) α_s2_-CN *f* (115–123) β-CN *f* (170–176) k-CN *f* (98–105)	Osteoblast proliferation activity	>10 ng/mL	[246].
	Chemical synthesis	VLPVPQK	β-CN *f* (170–176)	Antiapoptotic effect	>30 ng/mL	[247]
**Mare (equine)**	Koumiss	YQNPRLGPTGELDPATQPIVAVHNPVIV	β-CN *f* (217–241)	ACEI	14.53 μM	[15]
	Hydrolysis of whey with papain	NLEIILR TQMVDEEIMEKFR	β-LG *f* (71–77) β-LG *f* (143–155)	DPP-IV-inhibitory activity	86.34 μM 69.84 μM	[248]
	Milk	VAPFPQPVVPYPQR	β-CN *f* (176–189	Antioxidant	<500 μg/mL	[249]

n.s. = not specified; IC_50_: half maximal inhibitory concentration; MIC: minimal inhibitory concentration; nd: not determined.

## 5. Safety of Milk-Derived Bioactive Peptides

Commercialization of BPAs from milk proteins is still limited [193]. In addition to the lack of a cost-effective method of production, scarce information regarding their efficacy, safety, and bitter taste are the reasons heavily hindering their application in food and pharmaceutical sectors. Despite promising beneficial activities in humans, claims of BPAs are not supported by convincing evidence, appropriate clinical studies methodology, robust findings and, therefore, are usually rejected by the European Food Safety Authority (EFSA). In particular, human clinical trials should have an affordable experimental design including the use of appropriate control/placebo, eligibility criteria, and robust statistics to escape rejection of the obtained results. As regards BPAs as a supplement of food products, the EFSA also requests information on peptide quantity, peptide sequence and length, amino acid composition, molecular weight distribution, manufacturing process, physicochemical characteristics, and conditions of use. All of this combined with information on peptide bioavailability in humans, the understanding of mechanism of action in preclinical studies, and the safety of BPA administered in a chronic manner are areas in need of attention [193,250]. Overall, health claims of bioactive peptides can be divided into three types, “general function claims” (e.g., “Milk peptides help support the normal function of immune system”), “disease risk reduction claims” (e.g., “Egg peptides have shown to reduce cholesterol levels”), and “claims relating to children’s development or health” (e.g., fish peptides help children’s cognitive development). Researchers should submit an application to the EFSA through an EU country’s competent authority. After evaluation by the EFSAs’ Dietetic products, Nutrition and Allergies panel (EFSA NDA Panel), approval of a claim could be mainly based on the scientific substantiation and ability of an average consumer to understand the beneficial effects of the claims [251]. The application process usually takes from 6 to 8 months [252].

BAP safety is at the basis of clinical studies and food applications. Although their consumption is thought to be harmless, toxicological investigations must be increased. To date, no standard guidelines have been established for the safety assessment of milk-derived peptides, thus just a few investigations on BPA toxicity on humans have been undertaken. The toxicity of peptides on eukaryotic cells is usually measured by assaying their cytotoxicity or hemolytic activity on red blood cells before preclinical studies [192]. An in vitro work conducted by Ponstein-Simarro Doorten et al. [253] showed that daily ingestion of a hydrolysate derived from bovine milk (2 g/kg body weight) and containing the antihypertensive fragments IPP and VPP did not cause mutagenic or clastogenic effects [253]. Similarly, acute (2000 mg/kg) and daily (1000 mg/kg for 4 weeks) ingestion of casein hydrolysate [rich in ACEI RYLGY and AYFYPEL from αs1-casein fragments (90–94) and (143–149)] neither had any histological impact nor caused mortality in mice [254]. Also, intake of IPP-rich milk protein hydrolysates (7.5 mg IPP) for 1 month did not show adverse effects on hematology and clinical laboratory parameters with the exception of a significant increase in flatulence [199]. Other studies evaluating the toxicological aspects of milk-derived BPAs have been determined and the results to date suggest that they are safe [192].

Given the likelihood of the formation of allergenic peptides from food proteins, before their launch on the market, bioactive peptides should also be tested for allergenicity response. BAPs such as NSAEPEQSLC and VRTPGVDDGAL (from milk β-lactoglobulin) exhibit allergic reactions in humans. Similarly, the ACE inhibitor FFVAPFPE VFGK obtained from the casein hydrolysate was predicted to be an allergic peptide by the BIOPEP-UWM database [255], highlighting the need to provide allergenic warnings for products containing the related BAP.

## 6. The Role of Foodomics in the Detection and Characterization of Bioactive Peptides and Probiotics 

Foodomics has recently emerged as an innovative and promising discipline aiming at studying the relationship between food safety and quality and food nutrition and composition by complementing omics techniques such as proteomics, genomics, metabolomics and transcriptomics with chemometric and bioinformatics tools. It has expanded the application fields, proving to be a valuable approach to improve the food safety and quality of products for the health and well-being of consumers, and for a better understanding of the underlying mechanisms and pathways behind the development of the sensory and technological properties of foods [256,257,258]. Foodomics can help to improve the quality of dairy products [259] and in understanding technological processes of complex matrix requiring sophisticated technology able to extract large amounts of information for the detection of fraud and/or adulterations, microbiological safety, and the assessment and improvement of transformation industrial processes (e.g., fermentation and ripening). For a better and more exhaustive characterization of the complexity of the biological system of a food, omic technologies can be used at a multilevel mode, which is known as the multiomic approach, as demonstrated by its application in the control and quality improvement of milk-based products [259]. Due to the complexity of samples, no individual omic approach alone is able to unravel the complexity of the response and demonstrate a specific health claim of a potential probiotic formulation. To this aim, the multiomic approach and data integration are the novel frontiers for studying probiotics [260]. A system biology approach enables the integration of large datasets from experimental and theoretical models derived from genomics, metagenomics, transcriptomics and metatranscriptomics, proteomics, metabolomics, peptidomics and lipidomics, providing a comprehensive picture of the complex network of biological processes necessary to evaluate the beneficial effect of probiotic strains to the host in the treatment of specific diseases [261]. 

**Figure 1:**
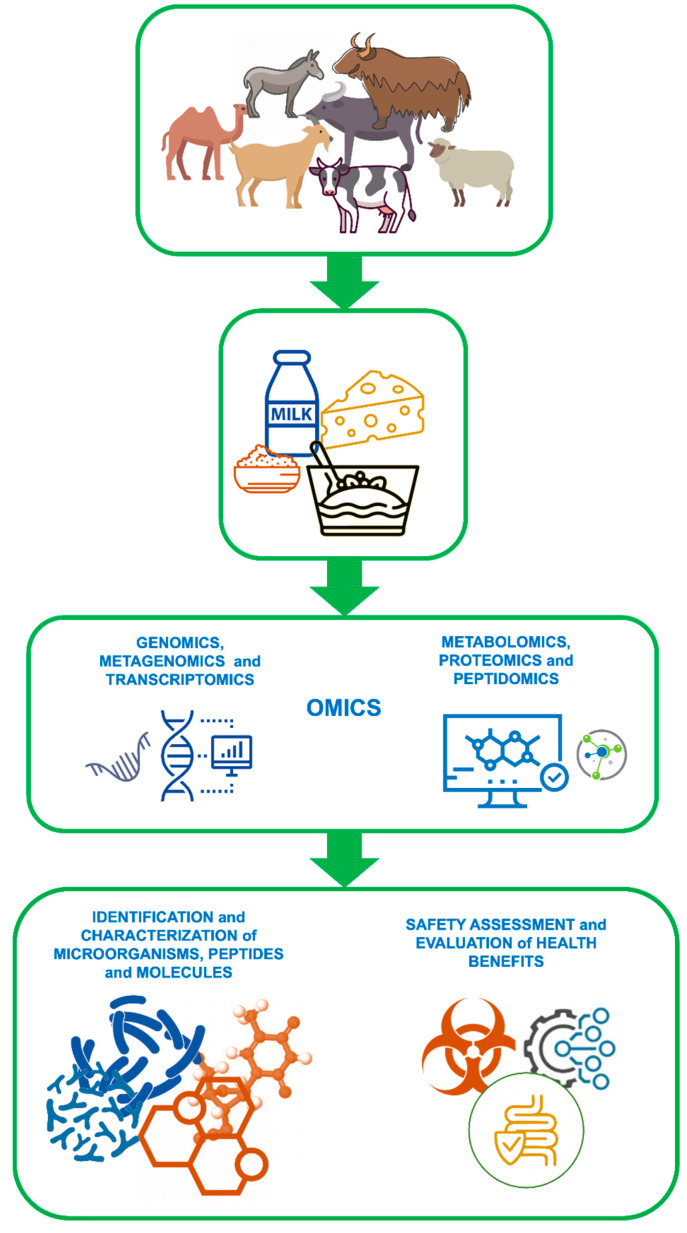
Schematic representation of the exploitation of omics technologies for the identification and characterization of probiotics and bioactive peptides from milk and dairy products.

These high-throughput approaches generating big data complemented with different cutting-edge analytical platforms, big data output, and bioinformatic tools have paved the way for a new concept of high-throughput analysis for elucidation of cellular mechanisms and pathways or for providing new knowledge on food safety and quality [262]. The utilization of such a multiomic approach generates data at different expression levels such as gene, transcript, protein, and finally metabolite. In this context, omics technologies target the expression of genes (genomes), transcription (transcriptomes) into mRNA, translation into proteins (proteomes) and secretion of metabolic by-products (metabolomes) within a specific sample. In Figure 1, the involvement of omics technologies in the identification and exploitation of probiotics and bioactive peptides is schematized.

### 6.1. Genomics and Transcriptomics

The first complete genome of a probiotic strain, the *Lactococcus lactis* ssp. *lactis* IL1403, was published in 2001 [263]. Since then, the contribution of omics technologies in the detection and characterization of probiotics strains has been pivotal and revolutionary, enabling the understanding of the biology of individual strains, deciphering the composition, evolution, dynamics, sensing and communication of complex microbial populations, their interaction with the host and activities related to their probiotic function [264].

Ventura et al. in 2009 [265] defined with the term propiogenomics the area of genomic-based studies of probiotic bacteria conferring health benefits to the host. The availability of hundreds of genomic sequences of potential probiotic strains and pan-genome analysis has enabled depicting the genetic signature of features associated with the probiotic functions: (i) the adaptation and colonization of the gut environment [266,267], which involved the presence of genes coding for human mucus-binding protein [268] conferring adherence capability and immunomodulatory effect, the presence of cell surface proteins, permeases, hydrolases and transporters [269]; (ii) salt and bile tolerance: the bile salt hydrolase (BSH) activity is responsible for the cholesterol-lowering effect of the probiotic strains. Survival in the low-pH environment of the gut is also important as a technological property, especially in the food sector, considering that yogurt and fermented milks are the most used carriers for probiotics administration. Despite the importance of efflux pumps and changes in the cell envelope [270,271,272], genomic analysis supported the identification of *arg*C, *arg*H, and *dap*A, as acid-tolerance-associated genes, which may play an essential role in high acid tolerance [273,274] in the probiotic *Bifidobacterium longum* NCIM 5672; (iii) antagonism against pathogens through production of antimicrobial compounds and short-chain lactic and fatty acids [275] with anti-inflammatory properties, gut barrier protection, and immunomodulation [276]. The bacteriocins are proteinaceous compounds that are able to damage the outer membrane of pathogenic bacteria, act as a protective barrier against biofilm development and have also been linked to probiotic immunomodulatory, anticancer, antioxidant, antibacterial, and cholesterol-lowering activities [277].

Genomic analysis also enables the preliminary in silico evaluation of the absence of undesirable traits, such as the presence of multidrug resistance or virulence genes [278,279,280,281]. This analysis is an essential requisite for pre-selection and utilization of strains in industrial formulation, which is regulated by the EFSA [282].

Furthermore, studying the genomes of probiotic microorganisms enables tracing the origin and evolution of strains and their relationship with gut microbiome, the genomic plasticity and the acquisition of genomic traits favoring their adaptation to the gut environment or the exertion of metabolic activities through horizontal gel transfer, such as in the *L. lactis* domestication [283].

The availability of the complete genomic sequences of probiotic microorganisms has contributed to the clarification of the correct taxonomy of a strain, which is pivotal to determining the authenticity of the probiotic authenticity [174,177], in particular in commercial broadly used strains (providing information for the development of genome-based methods to assess protocols to define the formulation of complex blends of multiple probiotics) [284]. To this aim, metagenomic approaches have been recently developed [285,286] and also combined with other omics technologies.

Metagenomic sequencing has enabled the taxonomic reconstruction of the human gut microbiome [287] and is crucial for evaluating the gut dysbiosis underlying diseases and the effect of probiotics administration on this ecosystem. 

In addition, while amplicon-based metagenomic analysis provides a complete picture of bacterial population in a particular food product, shotgun-based metagenomics is also able to provide knowledge related to the potential probiotic features embedded, which can be exploited by these products or by the microorganisms in them. 

Considering their potential role in modulating gut microbiota, this approach has recently been commonly used to characterize traditionally fermented foods, also with respect to safety aspects, such as the presence of antibiotic resistance genes. Kefir is one of the most studied milk-based fermented food, and recent works [288,289,290] applied metagenomics to evaluate the presence of probiotic species and predict their functionalities and identify isolates with antibacterial properties. A similar approach was used by Yasir et al. [291] to describe antimicrobial resistance genes in pasteurized and homemade fermented Arabian laban. Gioddu, which is the only Italian fermented milk product, was characterized by Maoloni et al. [292], who revealed a complex microbial population, dominated by *Lactobacillus delbrueckii*, but also including *L. kefirii*, and thus suggesting the presence of bioactive compounds similar to those of kefir.

Shangpliang et al. [293] applied a metagenomic approach to decipher the biomarkers for genes encoding different health-promoting functionalities in laal dadhi, an Indian fermented milk-based product. Genome mining of the metagenome-assembled genome of the five major abundant species (*Lactococcus lactis*, *Lactococcus chungangensis*, *Lactobacillus helveticus*, *Streptococcus thermophilus* and *L. delbrueckii*) enabled the identification of different probiotic and prebiotic functions, such as immunomodulation, short-chain fatty acid production, essential amino acid, antitumor genes, antimicrobial peptides, and vitamin biosynthesis.

### 6.2. Metabolomics

NMR- and MS-based technologies, particularly in the case of metabolomics, are essential tools for metabolome profiling [294]. Metabonomics is a subset of metabolomics and is defined as the quantitative measurement of the multiparametric metabolic responses of living systems to pathophysiological stimuli or genetic modification [295]; it can be considered as chemical profiling of a biological matrix (fluid, tissue, cell, etc.) since it aims at systematically profiling the widest range of small metabolites, ideally the complete collection (metabolome), present in that matrix, all this following an untargeted, comprehensive, and quantitative approach. UPLC-MS-based metabolomics has been exploited to track changes in the milk metabolomes during fermentation by two probiotic strains, *Lacticaseibacillus paracasei* PC-01 and *Bifidobacterium adolescentis* B8589 [296]. Such investigations aimed at analyzing probiotic-specific fermentative changes in milk providing information on the probiotic metabolism in that matrix and the potential beneficial mechanism of probiotic fermented milk, or investigating changes in the metabolites of fermented milk with novel probiotic *Lactobacillus plantarum* P9 [297]. The mentioned strategy can be applied to similar studies to characterize the fermentation process operated by probiotic strains in milk-based products. 

### 6.3. Proteomics and Peptidomics

Among the numerous technologies available, HPLC coupled to mass spectrometry (HPLC-MS) or capillary electrophoresis (CE) is very useful in peptidomics, proteomics, and metabolomics [298,299]. 

In proteomic studies, chromatography coupled with mass spectrometric detection enabled the identification of many bioactive peptides obtained in fermented milk and its hydrolysate thus proved to be a promising procedure in reducing analysis time and streamlining the whole process, skipping the traditional steps of peptide isolation and purification [300]. Proteomics is typically employed for qualitative analyses and characterization of protein/peptides [301] in the early detection of the foodborne parasite, etc. [302]. Proteomics is also an important and robust tool in nutrition and screening metabolic pathways. 

Metaproteomics is also a recent term and can be described as proteomics at the microbial level [303], and it is applicable in microbial studies for a better understanding of beneficial and spoilage or pathogenic microorganisms in foods under growing under different conditions [304]. Siragusa et al. [305] investigated the proteomics of *L. plantarum* in food materials providing some insights into growth behavior and microbial performance, making the adaptation of the strains possible in food biotechnology. De Angelis et al. [306] gathered numerous works in which the metabolic pathways, biotechnological characteristics, and interactions between different ecosystems of *Lactobacillus* sp., microorganisms widely used for the production of fermented meat, dairy and vegetables, are unveiled by using metaproteomics. The reconstruction of the metabolic pathways by means of bioinformatics may help in understanding these interactions. Combining metabolomics, transcriptomics, and proteomics as well as analyzing the resulting data enable a better understanding of the microbial metabolism underlaying the transformation of milk in its derivative products. 

Proteomics has been used in parallel with metabolomics to characterize the metabolism of different strains of *L. delbrueckii* subsp. *lactis* and *L. delbrueckii* subsp. *bulgaricus*, describing differentiated metabolic pathways for amino acids, folate, and sugar [133,307,308,309] and quantitatively detecting metabolites such as organic acids (e.g., acetate, lactate and formate) and fatty acids [310]. Metabolomic analysis has been used in numerous recent studies to characterize the activity of microorganisms involved in dairy biotechnology [311].

The recent development of ‘omics’ technologies has also enabled performing an investigation on the fate of food in the gastrointestinal tract and characterization of bioavailable food components. It has also provided a better understanding of the way that foods are metabolized by the human body [312]. More work will be necessary in the future to integrate current knowledge with the human physiological response upon food consumption [312]. 

Peptidomics is also gaining steadily increasing attention in the last two decades [313]. In the past, wet chemistry experiments and in vitro and in vivo characterization were conducted to identify and characterize BPAs, whereas mass spectrometry has been combined with machine learning to discover bioactive peptides nowadays [313]. In general, to circumvent the high cost for conducting in vitro and in vivo assays, bioinformatics is helpful in: (i) screening BPAs bioactivities, (ii) evaluating their bioavailability, (iii) exploring interaction mechanisms, (iv) assessing ADMET (absorption, distribution, metabolism, excretion, and toxicity) and allergenicity properties, etc. [314]. Although, bioinformatics has also highly progressed in this regard with newly developed models, algorithms, software, docking strategies and web servers, its potential use in the discovery of BPAs has yet to be fully explored. There is a broad spectrum of bioinformatics-aided procedures from BPA preparation to the evaluation of its bioavailability, bioactivity, allergenicity, taste and ADMET properties, which has contributed to the creation of bioactive peptide databases for efficiently retrieving in-depth information. To date, there are dozens of bioactive peptide databases available, each documenting one or more bioactivities (e.g., BIOPEP), so that users can gather a wide range of information (bioactivities, sources, articles, etc.) about a given peptide [315]. In addition, peptides are classified into different categories (source of origin, bioactivity, etc.), thus facilitating the data mining process before undertaking other bioinformatics studies (e.g., QSAR analysis).

## 7. Conclusions and Future Perspectives

Milk and its derivatives, above all fermented milk-based foods, have represented a main staple of the human diet throughout history and have gained considerable attention in recent years due to their health and nutritional benefits. Macro- and micronutrients provide nutritional properties, whereas BPAs and probiotics, which can be released during manufacturing processes or colonize milk and its derivatives, are responsible for the nutraceutical properties of these products. Several genera of probiotic bacteria such as bifidobacteria and lactobacilli have been isolated from milk and used to produce dairy products with a high standard of safety, quality and high nutritional and functional properties. Likewise, a long list of BPAs has been identified in native proteins from cow milk or milk from minor species; more from the latter have been obtained due to the efforts of recent studies aimed at discovering novel active sequences and developing innovative functional foods. Recent technologies in foodomics coupled with bioinformatic tools have efficiently contributed to gaining more insights into the structural elucidation of bioactive peptides and gaining an understanding of their functional properties as well as promoting and accelerating the understanding of host–probiotics/peptides interactions. This has contributed to revealing the genetic information, evolutionary relationship, physiological properties, metabolic network, and mechanism of action of probiotics and peptides. However, despite wide interest and the acquired knowledge, more investigations need to be performed for in-depth safety assessment and to elucidate the mechanisms of actions at the basis of the prevention or treatment of some diseases (allergies, metabolic disorders, etc.). Consequently, this summary of the current knowledge on probiotics and peptides from milk will help to further promote the intake of milk and derivatives produced from probiotic microbes able to improve the digestive health and overall nutritional well-being of consumers. Meanwhile, obstacles such as the need to implement efficient and cost-effective strategies for industrial-scale production, good manufacturing practices as well as well-designed clinical trials have been highlighted as challenges to be overcome in order to achieve commercialization for daily use in the human diet, on approval of the claims made.

## Figures and Tables

**Table 2:** Potential probiotic microorganisms isolated from dairy products (updated from Fusco et al., 2022 [34]).

Strain	Species ^a^	Source ^b^	Tests	Reference
832	*Saccharomyces* (*S.*) *cerevisiae*	Feta cheese (sheep milk)	In vitro cholesterol assimilation.	[96]
56 D4 D7 H 78 SR 82 SR41 SR246	*S. cerevisiae*	Danish blue veined cheese Gorgonzola, Italy Gorgonzola, Italy Cheese, Europe Spontaneous fermented cow milk, Sudan	In vitro probiotic characterization: acid and bile tolerance, adhesion to non-tumorigenic porcine jejunal epithelial cell line, cytokine assay.	[41]
BFE6058 BFE6059	*Lb. acidophilus* *Lb. acidophilus*	Kule naoto, Maasai fermented milk	In vitro probiotic and safety characterization: tolerance to acid and simulated GIT, antigenotoxic properties (ability to protect cells from DNA damage), hydrophobicity cholesterol assimilation, adhesion, bile salts deconjugation, auto-aggregation, biogenic amine production, DNAse activity, hemolysis, mucin degradation, antibiotic susceptibility.	[48]
F6	*Lb. fermentum*	Traditional dairy product in Inner Mongolia	In vitro probiotic characterization: tolerance to acid and simulated GIT, auto- and co-aggregation.	[97]
SM-B SM-1	*Lb. brevis* *Lb. plantarum*	Brazilian regional ovine cheese	In vitro probiotic and safety characterization: antioxidant activity, resistance to acid and bile salts, auto-aggregation, bile salt deconjugation, hydrophobicity, antagonistic activity, beta galactosidase activity, hemolysis.	[98]
AQ71	*E. faecium*	Azerbaijani Motal cheese (goat or sheep milk)	In vitro probiotic and safety characterization: antimicrobial activity, partial purification of bacteriocin, kinetics of bacteriocin production, detection of bacteriocin genes, acid and bile salt tolerance, antibiotic susceptibility, virulence genes detection, auto- and co-aggregation, enzymatic activity.	[99]
ZS07 K21	*Lb. plantarum* *Lb. plantarum*	Slovak Bryndza cheese (ewe milk)	In vitro probiotic and safety characterization: biogenic amine production, bile salt deconjugation, β-galactosidase activity, resistance to GIT, antibiotic susceptibility.	[100]
L10 L11 L13	*K. marxianus* *S. cerevisiae* *K. lactis*	Autochthon cheese starters	In vitro probiotic characterization: cell surface hydrophobicity, survival in simulated GIT, auto and co-aggregation with pathogens, antimicrobial activity.	[101]
Mb26	*Lb. plantarum*	Farmhouse goat’s milk cheese	In vitro probiotic and safety characterization: biofilm formation, bile salt hydrolase activity, resistance to HCl and oxgall, detection of plantaricin genes, production of hydrogen peroxide and bacteriocines, adhesion and inhibition of *L. monocytogenes* CECT 4032, production of biogenic amines, functional enzymatic activities, DNase activity, antibiotic susceptibility.	[102]
Three halotolerant lactobacilli (name of the strains not provided)	*Lb. plantarum*, *Lb. pentosus*, *Lb. acidipiscis*	Ripened Mexican tropical cheese (double cream Chiapas cheese)	In vitro functional characterization: bile salt deconjugation, antimicrobial activity, β-galactosidase activity, tolerance to simulated orogastrointestinal transit, adhesion to mucin, hydrophobicity.	[103]
FT259	*Lb. paraplantarum*	Brazilian semi-hard artisanal cheese	In vitro probiotic and safety characterization: tolerance to low pH and bile salts, antimicrobial activity, adhesion to Caco-2 cells, protease susceptibility and antilisterial activity of the antimicrobial compound produced by FT259; survival in simulated gastric juice, antibiotic susceptibility.	[104]
SJRP57	*Lb. delbruiekii* subsp. *bulgaricus*	Water-buffalo mozzarella cheese	In vitro probiotic and safety characterization: β-galactosidase activity, hydrophobicity, auto- and co-aggregation, tolerance, and effect of matrices on the survival under simulated GIT, adhesion and virulence genes detection, antibiotic and medicine susceptibility.	[105]
PRA205 PRA211 PRA172	*Lb. casei* *Lb. rhamnosus* *Lb. rhamnosus*	Ripened Parmigiano Reggiano cheese	In vitro probiotic and safety characterization: acid, bile salt and lysozyme tolerance, bile salts deconjugation, tolerance to simulated gastric and pancreatic juices, cell surface hydrophobicity, auto-aggregation, antibiotic susceptibility.	[106]
LP2	*Lb. fermentum*	Tulum cheese	In vitro probiotic and safety characterization: enzymatic activity, adhesion, survival in simulated GIT, antimicrobial activity, cholesterol assimilation, antibiotic susceptibility.	[107]
ZGPR3-18 BGGO5-47	*Lb. plantarum* *Lb. rhamnosus*	Croatian fresh soft cheese and Serbian white pickled cheese	In vitro probiotic and safety characterization: bile salt hydrolase activity, adhesion, cell surface hydrophobicity, survival in simulated GIT, assimilation of prebiotic substrates, cholesterol assimilation, capacity to modulate the immune response on GALT (gut associated lymphoid tissues) from rats, antibiotic susceptibility.	[108]
FS10 PM8	*Lb. rhamnosus* *Lb. paracasei*	Ragusano (cow milk) and Pecorino (sheep milk) Siciliano cheeses	In vitro probiotic and safety characterization: adhesion, hemolytic and bile salt hydrolase activities, tolerance to simulated GIT, hydrophobicity, antimicrobial activity, co- and auto-aggregation, antibiotic susceptibility.	[109]
SJRP55	*Leuc. mesenteroides*	Brazilian water buffalo mozzarella cheese	In vitro probiotic and safety characterization: auto and co-aggregation, adhesion, cell surface hydrophobicity, low pH, bile and NaCl tolerance, medication and antibiotic susceptibility, bile salts deconjugation, enzymatic activity.	[110]
ZIM 2408	*Kluyveromyces* (*K.*). *lactis*	Traditional cheese from Serbia and Croatia	In vitro probiotic characterization: adhesion, tolerance to simulated GIT, proliferation of gut-associated lymphoid tissue cells in the presence of non-viable yeast strain.	[111]
SJRP17	*E. durans*	cheese	In vitro probiotic and safety characterization: tolerance to NaCl, hydrophobicity, biogenic amine production, tolerance to simulated GIT, bile salt hydrolase activity, auto and co-aggregation, mucin degradation, virulence gene detection, medication and antibiotic susceptibility.	[112]
KJ722784	*Lb. plantarum*	Chhurpi (Indian dried cottage cheese)	In vitro probiotic characterization: cholesterol removal, exopolysaccharide production, tolerance to acid, auto-aggregation, lysozyme and bile salt, antimicrobial activity, cell surface hydrophobicity, β-galactosidase activity, hemolytic activity, antibiotic susceptibility.	[113]
KLDS 1.8701	*Lb. helveticus*	Chinese traditional cheese	In vitro probiotic characterization: antimicrobial activity, tolerance to simulated GIT.	[114]
1133 1086-1 1089 1138 1059 1141 1197	*Lb casei* *Lb. plantarum* *Lb. casei* *Lb. casei* *Lb. buchneri* *Lb. plantarum* *Lb. plantarum*	Tibetan qula (raw yak milk cheese)	In vitro probiotic and safety characterization: antimicrobial activity, acid and bile salt tolerance, antibiotic susceptibility, tolerance to simulated GIT, cell surface hydrophobicity.	[115]
B7	*Lb. plantarum*	Minas artisanal cheese	Protective effects of milk fermented by B7 on *Salmonella enterica* serovar Typhimurium infection in BALB/c mice (in vivo).	[116]
26 27 55 56	*Lb. plantarum*	Portuguese raw ewe milk semisoft cheese	In vitro antihemolytic, antimutagenic, anti-inflammatory, antioxidant and antimicrobial activities of bioactive peptides produced in the probiotic fermented milk.	[117]
SJRP30 SJRP145 SJRP146	*Lb. fermentum* *Lb. casei* *Lb. casei*	Water buffalo mozzarella cheese	In vitro probiotic and safety characterization: β-galactosidase activity, adhesion, aggregation and colonization factors, tolerance to simulated GIT, antimicrobial activity, antibiotic susceptibility, mucin degradation, detection of genes encoding adhesion, virulence, antibiotic resistance, and biogenic amine production.	[118]
LP4	*Lb. plantarum*	Minas artisanal cheese	In vitro probiotic and safety characterization: bile salt and artificial gastric juice tolerance, hydrogen peroxide production, antimicrobial susceptibility, antagonistic activity. In vivo protection against *Salmonella* Typhimurium infection in mice.	[119]
17bKL1 17bKlL2 NS2KL9 NS1KM2 14KM1 6688 KM	*K. lactis* *K. lactis* *K. marxianus* *K. marxianus* *K. marxianus* *K. marxianus*	Fiore sardo cheese (raw ewe milk)	In vitro probiotic and safety characterization: tolerance to GIT, bile salt hydrolase activity, hydrophobicity, auto-aggregation, adhesion to Caco-2 cells, hemolytic activity, susceptibility to antifungal agents, antimicrobial activity.	[120]
K5	*Lb. paracasei*	Feta-type cheese (sheep milk)	In vitro probiotic and safety characterization: antibiotic susceptibility and tolerance to simulated GIT.	[121]
DP3 DP21	*Lb. plantarum* *Lb. casei*	Iranian artisanal cheeses	In vitro technological, probiotic and safety characterization: bile salt and acid tolerance, auto-aggregation, cholesterol assimilation, adhesion to human intestinal cells, bile salt hydrolase activity, cell surface hydrophobicity, exopolysaccharide production, antagonistic activity, hemolytic activity, antibiotic susceptibility, monitoring of the pH and microbial survival of the probiotic fermented milk with added DP3 and DP21.	[122]
L3C1E8	*Lb. plantarum*	Pico cheese	In vitro probiotic characterization: low pH, bile salts and pancreatin tolerance, biofilm formation, adhesion, auto-aggregation, ability to inhibit the adhesion of *E. coli* ATCC 25922 to HT-29 cells, hydrophobicity. Production of conjugated linoleic acid from free linoleic acid.	[123]
J20 J23 J24 J25 J27 J28 J32 J37	*Lb. fermentum* *Lb. fermentum* *Lb. pentosus* *Lb. plantarum* *Lb. pentosus* *Lb. fermentum* *Lb. fermentum* *Lb. pentosus*	Cocido cheese	In vitro technological, probiotic and safety characterization: survival at pasteurization temperatures, survival under cold, lyophilizing, and freezing conditions, low pH and bile salt tolerance, hydrophobicity, auto-aggregation, mucin degradation, β-galactosidase activity, antibiotic susceptibility, hemolytic activity. In vivo study of the immunomodulatory effect of the probiotic fermented milk.	[124]
ACA-DC 2640 ACA-DC 4039 ACA-DC 264 ACA-DC 170	*Lb. plantarum* *Lb. plantarum* *Streptococcus* (St.) *thermophilus* *St. thermophilus*	Traditional Greek dairy products (Kasseri and Feta sheep milk cheeses)	In vitro adherence ability, antibacterial activity, and anti-inflammatory properties.	[125]
OB15	*E. faecalis*	Tunisian Rigouta cheese	In vitro probiotic and safety characterization: acid and bile salt tolerance, hydrophobicity, auto-aggregation, biofilm formation, adhesion to Caco-2 cells, gelatinase activity, hemolysis and cytotoxicity in Caco-2 cells, antibiotic susceptibility, virulence in the *Galleria mellonella* model, virulence gene detection.	[126]
SJRP38 SJRP43	*Lb. casei* *Lb. fermentum*	Water buffalo mozzarella cheese	In vitro probiotic characterization: survival of the probiotic strains and of milk fermented with the probiotic strains to simulated GIT, auto- and co-aggregation, hydrophobicity, β-galactosidase production, virulence genes detection.	[127]
DSM 32386	*Lb. brevis*	Alpine cheese	In vitro probiotic and safety characterization: γ-aminobutyric acid (GABA) production, Tolerance to pH, bile and pancreatic fluid, antibiotic susceptibility, antibiotic resistance gene detection.	[128]
FS103	*Lb. paracasei*	Sheep cheese	In vitro technological, probiotic and characterization: survival in the probiotic sheep and cow fermented milk during cold storage, tolerance to simulated GIT, acid and bile salt tolerance, antibiotic susceptibility, antagonistic activity.	[129]
PE24 PE25 PE44 PE61 PE85 PE86	*Lb. paracasei* *Lb. rhamnosus* *Lb. rhamnosus* *Lb. rhamnosus* *Lb. paracasei* *Lb. paracasei*	Piacentino Ennese PDO cheese	In vitro probiotic and safety characterization: survival in simulated GIT, acid, lysozyme and bile salt tolerance, hydrophobicity, antimicrobial activity, anti-inflammatory activity, adhesion, auto- and co-aggregation, antioxidant activity, preliminary identification of metabolites responsible for antagonistic activity against pathogens, virulence and antibiotic resistance genes detection, biogenic amine production, DNAse, gelatinase, hemolytic and mucin degradation, antibiotic susceptibility and MIC determination.	[130]
C1Lb21 GiLb5 G4Lb7	*Lb. brevis* *Lb. plantarum* *Lb. pentosus*	Serpa Cheese (soft cheese)	In vitro probiotic and safety characterization: aggregation activity, tolerance to simulated GIT, cell surface hydrophobicity, growth on prebiotic, short-chain fatty acid (SCFA) production, biogenic amine production, antibiotic susceptibility.	[131]
E297	*E. faecium*	Minas Frescal cheese	In vitro probiotic and safety characterization: survival in simulated GIT, gelatinase, antimicrobial resistance, virulence and antibiotic resistance genes detection, lipase and DNase production, hemolysis.	[132]
A6	*Lb. brevis*	Minas artisanal cheese	In vitro probiotic and safety characterization: bile salts and artificial gastric juice tolerance, hydrogen peroxide production, antimicrobial activity, antibiotic susceptibility.	[133]
PMD74	*E. lactis*	Ezine cheese (made from ewe, cow and goat milk)	In vitro functional and safety characterization: bile salt hydrolysis (BSH) and mucin degradation activity, survival in simulated GIT, antimicrobial activity, antibiotic susceptibility, hemolytic and gelatinase activity, amino acid decarboxylase detection, detection of virulence genes.	[134]
B7 D1	*Lb. plantarum* *Lb. rhamnosus*	Minas artisanal cheese	In vivo (in mice) and in vitro functional characterization: acid and bile salt tolerance, antimicrobial susceptibility.	[135]
SMVDUDB2	*Pediococcus acidilactici*	Kalarei (traditional cheese)	In vitro probiotic and safety characterization: exopolysaccharide production, auto and co-aggregation, tolerance to simulated GIT, hydrophobicity, antagonistic activity, antibiotic susceptibility.	[136]
L3C21M6	*Lb. paracasei*	Artisanal Pico cheese	In vitro probiotic and safety characterization: acid, bile salts and pancreatin tolerance, bile salts deconjugation, adhesion, virulence genes detection, antibiotic susceptibility. In vitro cholesterol-lowering activity and ability to degrade histamine.	[137]
OSY-EGY	*E. durans*	Egyptian artisanal cheese	In vitro probiotic and safety characterization: resistance to low pH and bile salts, auto-aggregation, adhesion, hydrophobicity, cholesterol-lowering activity, antioxidant effect, antimicrobial activity, heamolysis, gelatinase production, cytotoxicity in human colorectal adenocarcinoma cell line Caco-2 cells, antibiotic susceptibility, virulence genes detection.	[138]
KMJC4 KMJC1	*Lb.plantarum* *Lb. brevis*	Iranian Jug cheese (made from cow milk or a mixture of sheep and cow milks)	In vitro functional and safety characterization: acid and bile salt tolearance, survival in simulated GIT, adhesion capacity, antimicrobial activity, heamolysis, antibiotic susceptibility.	[139]
Not specified	*Pediococcus acidilactici*	Wara, Nigerian unripened soft cheese	In vitro probiotic and safety characterization: acid and bile salt tolerance, survival in simulated GIT, antimicrobial activity, auto-aggregation, adhesion to hydrocarbon assay, exopolysaccharide production, heamolytic activity.	[140]
Os4 Kor14	*Lb. plantarum* *Lb. plantarum*	Polish regional cheeses	In vitro technological and probiotic characterization: enzymatic profile, adhesion, tolerance to simulated GIT, anti-staphylococcal activity, antibiotic resistance. Assessment of the anti-staphylococcal activity in the probiotic skim milk.	[141]
B9, B13 and B38	*Lb. brevis*	Algerian homemade cheeses made from either cow, sheep or goat milk	In vitro probiotic and safety characterization: tolerance to simulated GIT, hydrophobicity, adhesion capacity, auto and co-aggregation, antimicrobial activity, bile salt deconjugation, in vitro cholesterol, lowering ability, antioxidant activity, antibiotic susceptibility.	[142]
B14	*L. acidophilus*	Iranian cheese	In vitro functional and safety characterization: acid and bile salt tolerance, resistance to simulated GIT, cell surface hydrophobicity, auto- and co-aggregation, adhesion capacity, antibiotic susceptibility, hemolytic activity, biogenic amine production.	[143]
RI53 RI42	*E. faecium*	Ahvaz semi-skimmed natural cheese Ardabil full-fat natural cheese	In vitro probiotic and safety characterization: aggregation properties, acid and bile salt tolerance, survival in simulated GIT, production of exopolysaccharides, antimicrobial activity, antibiotic susceptibility.	[144]
5C 1D 9 KE 3 TB 7 KB 3B	*E. faecalis* *E. hiriae* *E. durum* *E. faecium* *E. faecalis*	Motal cheese (traditional raw milk Iranian cheese)	In vitro functional and safety characterization: survival under simulated GIT, adhesion to Caco-2 cells, antibacterial activity, auto- and co-aggregation, cholesterol assimilation, hemolysis, antibiotic susceptibility, virulence gene detection.	[145]
1QB77	*Lb. plantarum*	Brazilian artisanal cheese	In vitro probiotic and safety characterization: auto-aggregation, low pH and bile salt tolerance, hydrophobicity, tolerance to simulated GIT, adhesion, biogenic amine genes detection, antagonistic activity, antimicrobial properties of the potential probiotic strain in microscale cheeses.	[146]
S1113 S104 S1121 S202	*E. faecium* *E. durans* *E. durans* *E. durans*	Artisanal white cheeses from Turkey	In vitro probiotic and safety characterization: antibiotic susceptibility bile salts and simulated gastric juice tolerance, antimicrobial activity.	[147]
894	*E. faecium*	Turkish Tulum cheese	In vitro probiotic and safety characterization: auto-aggregation, antibacterial activity, bile and simulated gastric juice tolerance, cell surface hydrophobicity, cholesterol removal, β-galactosidase activity, exopolysaccharide production, antibiotic susceptibility, hemolytic activity, biogenic amine production.	[148]
SIM12 SIS16 1734 Lh43	*Lb. helveticus*	Italian hard cheeses	In vitro assessment of the immunomodulatory properties (cytokine production in culture media and in cheese). In vitro probiotic characterization: proteolytic, peptidase, antioxidant and β-galactosidase activities, ability to produce folate.	[149]
MT1-MT6	*Lactip. plantarum*	Ten different traditional Iranian cheeses produced either from cow, ewe or goat milk	In vitro probiotic and safety characterization: resistance to acid, bile salts and simulated GIT, antibiotic susceptibility, antibacterial activity.	[150]
T40	*L. paracasei*	Tenate cheese	In vitro probiotic characterization: resistance to acid and bile salt, antimicrobial activity.	[151]
SJ14	*Lactp. plantarum*	Algerian traditional cheese “Jben”, made from with raw cow, goat, or sheep milk	In vitro probiotic and safety characterization: acid and bile salt tolerance, antibacterial and antifungal activities, exopolysaccharides production, adhesion to Caco-2 cells, antibiotic susceptibility.	[152]
12 not typed isolates	Isolates of the *Enterococcus* genus by phenotypic tests	Labneh Anbaris (goat milk cheese)	In vitro functional characterization: acid and bile tolerance, antibacterial activity, ACE inhibitory activity.	[153]
AD73	*L. plantarum*	Milk kefir	In vitro probiotic characterization: adhesion to Caco-2 cells, antibacterial activity, acid and bile salt tolerance, cytotoxic activity in Caco-2 cells, survival in lab-scale production of probiotic Chevre cheese.	[154]
14 15 16 47 60 70	Not typed isolates that were identified by biochemical methods as *Lactobacillus* spp.	Ethiopian spiced cottage cheese	In vitro probiotic and safety characterization: hydrophobicity, acid and bile salt tolerance, antimicrobial activity, hemolysis, antibiotic susceptibility.	[155]
9 strains isolated from rala 3 strains isolated from pingo	*Yarrowia lipolytica* *Kodamaea ohmeri*	Endogenous ferment (*pingo*-whey or *rala*-grated ripened cheese) of artisanal Minas cheese	In vitro probiotic and safety characterization: acid and bile tolerance, auto-aggregation, hydrophobicity, antibiotic susceptibility, co-aggregation with pathogens, antimicrobial activity.	[156]
54B, 54C, 55A, 55B, 95E	*Lb. plantarum* *Lb. plantarum* *Lb. plantarum* *Lb. pentosus* *P. pentosaceus*	Ethiopian spontaneously fermented cheese	In vitro probiotic and safety characterization: survival in simulated GIT, antimicrobial activity, in vitro immunostimulatory activity, antibiotic susceptibility.	[157]
4R15 9N2 3R2	*Lb. paracasei*	Tulum cheese (Turkish goat milk cheese)	In vitro probiotic and safety characterization: bile salt hydrolase activity, tolerance to simulated GIT, antimicrobial activity, adhesion to HT-29 cells, biogenic amine production, antibiotic susceptibility.	[158]
B15 BM10 C30	*Lb. plantarum*, *Lb. casei*, *E. durans*	Bouhezza cheese (made from raw goat or sheep milk) Bouchezza cheese Goat’s butter	In vitro probiotic and safety characterization: phenol, acid and bile salt tolerance, hydrophobicity, auto- and co-aggregation, antimicrobial activity, exopolysaccharide production, hemolysis, antibiotic susceptibility.	[159]
CFS	*P. acidilactici*	Iranian cheese	In vitro probiotic and safety characterization: NaCl, acid and bile salt tolerance, antimicrobial activity, in vitro anticancer assessment, antibiotic susceptibility.	[160]
LAB-2 LAB-3 LAB-6	*Lb. rhamnosus* *Lb. rhamnosus* *E. durans*	Egyptian fermented dairy samples	In vitro probiotic and safety characterization: acid and bile salt tolerance, antimicrobial activity, antibiotic susceptibility.	[161]
16 26 28 29 21	*P. pentosaceus* *Leucc. mesenteroides* *Lb. casei* *Lb. fermentum* *E. faecium*	Colombian double creame cheese	In vitro probiotic and safety characterization: acid and bile salt tolerance, auto-aggregation, hydrophobicity, exopolysaccharide production, antimicrobial activity, hemolysis, antibiotic susceptibility.	[162]
L11 L13 L33	*Lb. pentosus* *E. faecium* *Lb. plantarum*	Iranian cheese	In vitro probiotic and safety characterization: hydrophobicity, auto- and co-aggregation, antimicrobial activity, hemolysis, antibiotic susceptibility, biogenic amine production.	[163]
BD3 BR4 MR2	*Lb. paracasei* *Lb. plantarum* *Lb. fermentum*	Egyptian cheeses	In vitro probiotic and safety characterization: resistance to gastric acidity and bile salts, exopolysaccharide production, auto-aggregation, hydrophobicity, bile salt hydrolysis, hemolysis, antibiotic susceptibility.	[164]

^a^ The genus *Lactobacillus* has been reclassified into 25 genera [94]. In this table, the old and the new nomenclature are provided. ^b^ Where not specified, the milk used for the production of dairy products is cow’s milk.

## Data Availability

Data is contained within the article.
